# Escape to Alcatraz: evolutionary history of slender salamanders (*Batrachoseps*) on the islands of San Francisco Bay

**DOI:** 10.1186/1471-2148-9-38

**Published:** 2009-02-11

**Authors:** Iñigo Martínez-Solano, Robin Lawson

**Affiliations:** 1 Museum of Vertebrate Zoology, University of California, Berkeley, 3101 Valley Life Sciences Building, Berkeley, CA 94720-3160, USA; 2 Ecology and Evolutionary Biology, University of Connecticut, Storrs, 75 N Eagleville Rd., Unit 3043, Storrs, CT 06268-3043, USA; 3 Instituto de Investigación en Recursos Cinegéticos (Universidad de Castilla la Mancha-CSIC), Ronda de Toledo, s/n, 13005 Ciudad Real, Spain; 4 Department of Herpetology and Center for Comparative Genomics, California Academy of Sciences, 55 Music Concourse Drive, Golden Gate Park, San Francisco, CA 94118-4503, USA

## Abstract

**Background:**

Island populations are excellent model systems for studies of phenotypic, ecological and molecular evolution. In this study, molecular markers of mitochondrial and nuclear derivation were used to investigate the evolution, structure and origin of populations of the California slender salamander (*Batrachoseps attenuatus*) inhabiting the six major islands of San Francisco Bay, formed following the rising of sea level around 9,000 years ago.

**Results:**

There was a high degree of congruence in the results of analyses of nucleotide and allozyme data, both of which strongly support the hypothesis that, for the majority of the islands, salamanders are descended from hilltop populations that became isolated with the formation of the Bay ca. 9,000 years ago. There are two exceptions (Alcatraz and Yerba Buena) where the evidence suggests that salamander populations are wholly or in part, the result of anthropogenic introductions.

Comparison of the molecular data and the interpretations drawn therefrom with an earlier morphological study of many of the same salamander populations show some of the same evolutionary trends.

**Conclusion:**

In spite of marked differences between the evolutionary rates of the two kinds of molecular markers, both indicate distinctive and similar patterns of population structure for *B. attenuatus *in the San Francisco Bay Area and its islands. With the two noted exceptions, it is clear that most island populations were established prior to the 9,000 years since the formation of the Bay. Results of coalescence-based analyses suggest that for most island populations the mtDNA lineages from which they were derived date from the Pleistocene.

It can be said that, based on observed values of genetic diversity, the last 9,000 years of evolution on these islands have been characterized by relative stability, with the occasional extinction of some haplotypes or alleles that were formerly shared between island and mainland populations but overall maintaining high levels of variation (with the exception of Alcatraz). In contrast, there is some evidence for rapid morphological changes between populations in some islands and their closest mainland counterparts. This pattern of rapid morphological divergence (e. g., resulting from founder effects) is similar to that observed in other studies about recent colonization of island habitats.

## Background

Islands have long been the subjects of both theoretical and empirical studies in Evolutionary Biology. They are often regarded as "natural laboratories" for the study of speciation because geographic isolation promotes morphological, ecological and genetic divergence of newly established populations with respect to their mainland counterparts and thus they offer an excellent opportunity to identify key factors in species formation. In more recent times, the use of molecular markers has added impetus to the analysis of the complex processes involved in differentiation of island populations. The study of colonization patterns and the effects of isolation on divergence may allow identification of potential source populations either from the mainland or other islands in a stepping stone or sweepstake model of colonization. Characterization of patterns of gene flow, with the addition of a temporal component, especially in cases when geological information is available allows dating with some precision the amount of time passed since populations have been isolated.

The islands of the San Francisco Bay (California, USA) constitute an interesting system from an evolutionary perspective because their origin is relatively recent (Holocene) and thus their study might provide clues to understanding the consequences of population isolation after the interruption of gene flow following the rising of the sea level, especially in a short time scale (the last 9,000 years). The islands have been studied from different perspectives, including detailed geological, botanical and faunistic surveys [1]. We note here that since the early days of the European settlement of California, each of the islands in our study region has been impacted by human activities either by mining and/or building operations. This has resulted in the establishment on the islands of many non-native invertebrates, these are exemplified by anthropochorus terrestrial isopods of European origin where one to three species are flourishing on each of the islands with the possible exception of Red Rock Island [2]. In the case of the latter island the robust population of the native scorpion *Uroctonus mordax *may have prevented the establishment of terrestrial isopods. However, on the remaining islands where these isopods form a substantial part of the slender salamander diet (pers. obs.) their presence is likely to have had a positive effect on salamander populations.

Among the faunal surveys of the San Francisco Bay islands, the salamanders in particular have been the subjects of thorough studies, including the description of morphological variation and the collection of extensive demographic data on these island populations [3]. This study revealed an extraordinary variability within and between islands as well as differences with respect to their closest mainland counterparts in both ecological and morphological characters. However, a detailed genetic study of the salamander populations on these islands, which would provide a sound background from which to view those previous studies and which would aid in producing robust hypotheses of the evolutionary processes generating those patterns, has been lacking.

Of the two salamander species present on the islands of the San Francisco Bay area, the most abundant is the California slender salamander (*Batrachoseps attenuatus*). Dispersal is very rare in this salamander, which has some of the lowest reported home ranges in vertebrates and exhibits strong phylopatry, promoting isolation by distance. Anderson [3] advanced two hypotheses to explain the presence of salamander populations on the San Francisco Bay islands. These two hypotheses can be tested by means of data gathered from discrete molecular markers:

1) Populations were continuously distributed before sea levels rose 9,000 years ago and were subsequently isolated as hills or mountaintops became islands. In this case, we would expect to find haplotypes that are exclusively found on islands and similar values of genetic diversity on mainland and island populations.

2) Island populations were established more recently, after sea levels rose, by rafting or by anthropogenic introduction. In this case, haplotypes found on islands would include a subset of those found in nearby mainland populations and levels of genetic diversity would be lower on islands due to founder effects.

Of course, each island constitutes a different "experiment", and test results may favor one or the other hypothesis, or combinations of both in different islands. For instance, a third possibility would be that populations were present before sea levels rose and were thereafter augmented by occasional immigrants by rafting. However, with the data at hand it is problematic to discriminate between cases where we find a mixture of exclusive (island) and widespread (mainland) haplotypes on islands because of incomplete sorting of polymorphisms (expected when population divergence times are recent, as in our example) and cases where the same pattern is caused by a long history of isolation followed by occasional immigration introducing mainland genetic variants in island populations.

A previous detailed phylogeographic study of *B. attenuatus *[4] provides an historical context in which to analyze the evolution of island populations of *B. attenuatus *in the study region, including their most plausible evolutionary origin. This phylogeographic study indicated that populations are geographically structured and exhibit a significant pattern of isolation by distance, with populations in geographic proximity generally exhibiting greater genetic similarity, except in areas of narrow parapatry where some of the five well-differentiated mtDNA lineages identified in that study come into secondary contact. Several of these mtDNA lineages within *B. attenuatus *coexist in the vicinity of the San Francisco Bay, making more detailed examination necessary to understand the origin and evolutionary affinities of the island populations. In this paper, we use mitochondrial DNA (mtDNA) and allozyme data (genetic data of nuclear origin) to test hypotheses about the origin of island populations and to reconstruct their evolutionary history. These results are then used to discuss the patterns of morphological differentiation between islands and between island and mainland populations described by Anderson [3].

## Methods

### Sampling

**Figure 1:**
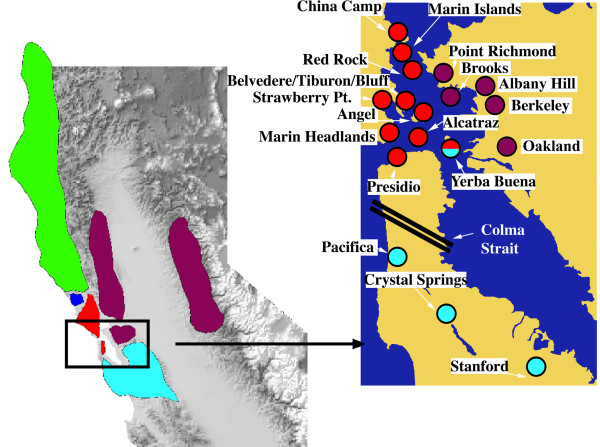
**Sampling and mtDNA haplogroups in *B. attenuatus***. Map of California showing the distribution of mtDNA lineages in *B. attenuatus *(colors) (left). Inset (right) shows populations sampled in the San Francisco Bay Area, with colors also indicating mtDNA haplotypes found in each case. The parallel black lines indicate the approximate location of the Colma Strait (see text). See Figure 3 for more details.

We compiled two independent, complementary datasets to investigate the evolution of populations of *B. attenuatus *on the islands of San Francisco Bay. Both of them include samples from all islands studied originally by Anderson [3] (Fig. 1): Angel (area = 445.1 hectares -ha-), Yerba Buena (78.1 ha), Brooks (26.3 ha), East (11.7 ha) and West (1.2 ha) Marin, and Red Rock (2.3 ha), as well as Alcatraz island (7.6 ha), which was not studied by Anderson [3]. For West Marin island, only allozyme data was available for analyses. Details of collecting localities and sample sizes for mtDNA can be found in Table 1 and Additional file 1.

**Table 1:** Genetic variability in selected populations in the mtDNA dataset.

Population	n	s	n. haplotypes	exclusive haplotypes	Haplotype diversity	Nucleotide diversity	k
China Camp *	15	8	6	3	0.800	0.00385	2.610
East Marin *	29	21	8	5	0.650	0.00573	3.882
Red Rock	7	1	2	2	0.286	0.00042	0.286
Belvedere	8	7	6	3	0.893	0.00348	2.357
Tiburon	10	3	3	1	0.511	0.00112	0.756
Bluff Point	9	3	2	0	0.222	0.00098	0.667
Strawberry Point	10	20	5	3	0.822	0.00653	4.442
M. Headlands	19	13	11	10	0.901	0.00287	1.942
Angel +	80	11	11	10	0.658	0.00147	0.997
Alcatraz +	30	0	1	0	0.000	0.00000	0.000
Yerba Buena #	22	38	4	2	0.455	0.01984	13.429
Presidio #	39	5	2	0	0.189	0.00140	0.945
Brooks §	19	6	5	3	0.731	0.00242	1.637
Albany Hill §	20	7	6	4	0.674	0.00303	2.053

n = sample size; s= number of segregating sites; k = average number of nucleotide differences. The symbols (*, +, # and § indicate localities that share haplotypes (see text for details).

### mtDNA

Partial sequences of the mitochondrial cytochrome *b *gene were used to analyze patterns of genetic variation at different spatial scales in *B. attenuatus*. Details of DNA extraction, amplification and sequencing procedures are reported in a previous paper [4]. The final dataset included sequences from 711 individuals, of which 178 were already reported in that study and 94 were downloaded from GenBank (Accession Numbers: DQ348971–DQ349064). The remaining 439 sequences were generated for this study and are also deposited in GenBank (Accession Numbers: FJ417408–FJ417846).

Standard estimates of genetic variability in selected island and mainland populations, including number of segregating sites, haplotypic diversity, nucleotide diversity and average number of nucleotide differences within populations were estimated with the software package DNASP [5].

At the broadest spatial scale, phylogenetic analyses were used to identify the main mtDNA lineages within *B. attenuatus *and to investigate the phylogenetic affinities of island populations (i.e., assign haplotypes found on islands to the main mtDNA lineages identified in a previous study [4]). Sequences were collapsed to unique haplotypes with the computer program Collapse (D. Posada, available at ) prior to phylogenetic analyses, which were based on Maximum Parsimony (MP), Maximum Likelihood (ML) and Bayesian inference as implemented in the software packages PAUP, GARLI and MrBayes v3.1.2 [6-8].

Because haplotypes found on islands did not form monophyletic groups in phylogenetic analyses, we further investigated population history by inferring population (rather than gene) trees in the Southern_N clade, the most widespread in the study area. We used the "minimize deep coalescences" method [9] as implemented in Mesquite 2.01 [10] to recover the population tree that minimizes the number of deep coalescences in the gene tree [11]. For this analysis, we used as the preferred gene tree a ML tree estimated with PAUP with populations in ten predefined groups based on geographic proximity. Each island conformed a group, whereas for mainland samples an arbitrary radius of three miles was used to delimit group samples.

Finally, in order to estimate the temporal scale associated with the evolutionary history of island populations, we estimated the time to the most recent common ancestor (TMRCA) of selected groups of haplotypes. These groups include the five main mtDNA lineages identified in a previous study [4], reference mainland populations as well as haplotypes found exclusively on islands. In the latter case, TMRCAs represent a minimum colonization age for the islands. When haplotypes occurring in mainland populations were also found on islands, we estimated the TMRCA of those mainland + island haplotypes in order to obtain further insight into the time of colonization. In this case, TMRCAs represent a maximum time boundary for island colonization. It must be kept in mind that TMRCAs estimate gene divergence and thus provide overestimates of population divergence, which are necessarily more recent. The magnitude of this difference cannot be assessed with single-locus methods, but it is the method of choice for recent divergence time estimates [12]. The software BEAST 1.4.8 [13] was used to estimate TMRCAs. We used a GTR+I+G model of evolution on an un-partitioned dataset, and implemented an uncorrelated lognormal relaxed molecular clock method [14]. We used a normal distribution with a mean of 0.0075 and a standard deviation of 0.0025 as a prior for mutation rate in cytochrome *b *to reflect *a priori *uncertainty in this parameter based on the values reported by Mueller [15]. We also used a lognormal prior for the root height parameter to incorporate information from the fossil record (fossils attributed to *Batrachoseps *dating from 5.3 million years ago have been recovered in the vicinities of the San Francisco Bay Area [16], thus providing a minimum time estimate for the parameter root.height in BEAST analyses). Six independent runs of different lengths and totaling 120 million generations were performed and subsequently combined in LogCombiner (distributed as part of the BEAST software package) to check for convergence of the posterior distributions of parameters of interest and calculate 95% confidence intervals for selected TMRCAs.

### Allozymes

Approximately 1 to 2 mm of tissue excised from the tail tip of the salamanders was mechanically homogenized in an estimated five volumes of grinding buffer composed of 0.25 M sucrose containing 10 mM mercaptoethanol, 0.001 mM EDTA, 0.1 M MgCl_2 _and 0.01 M Tris adjusted to neutrality with hydrochloric acid. Tissue homogenates were stored frozen until needed for electrophoretic assay.

For electrophoresis we used a standard horizontal starch-gel system [17-19]. The gene products of a total of 21 enzyme-coding loci were assayed for electrophoretic variability, the loci scored are given in Additional file 2. Two electrophoretic buffer systems were used. For Lactate dehydrogenase we used the discontinuous Tris-citrate-borate system [20] and for the remaining enzymes we used a modification of the citrate-aminopropyl morpholine buffer [21] adjusted to pH 8.0. Electromorphs were rendered visible for scoring using staining methods [22,23]. Designation of loci followed Manchenko [24]. Alleles (electromorphs) were designated alphabetically generally starting with the most common.

A total of 1148 individuals from 14 populations were scored at 21 loci. Estimates of observed and expected heterozygosity (H_O _and H_E_), number of private alleles, and allelic richness per population were calculated with the software package Biosys-1 [25]. Tests of linkage disequilibrium and Hardy-Weinberg equilibrium were calculated at each locus and population with Genepop [26].

We tested for genetic structure in the allozyme dataset with three methods. First, we estimated pairwise F_ST _values [27] and their statistical significance in Arlequin version 2000 [28]. Additionally, we calculated Nei's genetic distances [29] with Biosys-1 and represented these values on a bi-dimensional space through multidimensional scaling [30] using the software package GenAlEx [31]. Finally, we used the software "Structure" 2.2 [32] to infer the optimal number of genetic clusters in our dataset. Structure assumes Hardy-Weinberg equilibrium and uses Bayesian algorithms to infer the assignment probability of any given individual to each genetic cluster based on allele frequencies. It also identifies the number of genetic clusters (*K*) with a highest posterior probability without taking into account prior information on the number of sampling localities. We used an admixture model with correlated allele frequencies for values of *K *ranging from one (panmixia) to eight clusters, and performed five runs for each value of *K*, with a "burn-in" period of 500,000 iterations and posterior searches of 1,000,000 MCMC iterations. Individuals with 10% or more missing data were excluded from the analyses, which included 949 individuals from the 14 populations sampled.

## Results

### mtDNA

In the islands of San Francisco Bay we found mtDNA haplotypes corresponding to three of the five mtDNA lineages identified in Martínez-Solano *et al*. (2007) [4]. The remaining two lineages occur naturally only north of the current study area and so their presence is not expected. The most widespread of the lineages found in the Bay Area is the Southern_N clade, present in the islands of East Marin, Red Rock, Angel, Alcatraz and Yerba Buena. On Yerba Buena Island, salamanders with haplotypes from the Southern_S clade were found in sympatry with those having haplotypes of the Southern_N clade in both of the two localities sampled on the island. This is the only instance so far identified over the range of *B. attenuatus *where salamanders of two of the haplotype clades have been found in sympatry. Finally, samples from Brooks Island grouped with Martínez-Solano *et al.'*s (2007) [4] Eastern mtDNA clade (Fig. 1).

In general, levels of mtDNA variability are high in all islands studied, with unique haplotypes found on each island with the exception of Alcatraz, where only one haplotype was found. This haplotype also occurs in the Angel Island population. With this exception, all other haplotypes found on Angel Island and all from Red Rock are unique to these two islands, whereas in the remaining islands we found a mixture of exclusive island haplotypes and those shared with mainland populations (Table 1). For example, on East Marin we found haplotypes that are also found in China Camp, on Yerba Buena there were haplotypes also found in the populations of Pacifica and Presidio and on Brooks island we found haplotypes that are also present in the populations of Point Richmond, Albany Hill, Point San Pablo, Berkeley and Oakland. Levels of haplotype diversity range from zero on Alcatraz to 0.901 in the mainland population of Marin Headlands. Low diversity values are found in the populations of Presidio (mainland) with 0.189, only two haplotypes in 39 individuals sampled; Bluff Point (mainland) with 0.222, two haplotypes in the nine specimens sampled; and Red Rock with 0.286, two haplotypes in seven individuals sampled. The highest values of haplotype and nucleotide diversity were always found in mainland populations adjacent to the west shore of the North Bay (Marin Headlands, China Camp, Belvedere and Strawberry Point, Figure 1 and Table 1). Pairwise values of uncorrected genetic distances as well as geographic distances between populations are presented in Table 2.

**Table 2:** Genetic differentiation between populations based on mtDNA data.

	C. Camp	E Marin	R. Rock	Tiburon	M. Headlands	Y. Buena	Alcatraz	Angel	Presidio	Stanford	C. Springs	Brooks	A. Hill
China Camp	*	**5**	10	15	21	24	21	17	23	68	44	17	20
E Marin	0.006	*	5	10	17	19	16	12	18	63	39	12	16
R. Rock	0.007	**0.007**	*	**7**	15	14	12	8	14	58	35	8	12
Tiburon	0.007	0.008	0.003	*	8	10	6	**3**	8	54	28	10	14
M. Headlands	0.032	0.030	0.028	0.030	*	12	7	8	4	51	24	15	19
Y. Buena	0.050	**0.051**	**0.046**	0.047	0.053	*	5	8	**9**	44	22	9	11
Alcatraz	0.028	**0.028**	**0.024**	0.025	0.010	**0.044**	*	4	**5**	48	23	10	13
Angel	0.028	**0.029**	**0.024**	0.026	0.013	**0.045**	**0.003**	*	7	51	27	7	11
Presidio	0.032	0.032	0.028	0.029	0.014	0.046	0.004	0.007	*	47	21	14	18
Stanford	0.056	0.055	0.050	0.052	0.060	0.026	0.054	0.054	0.055	*	29	52	51
C. Springs	0.056	0.056	0.051	0.052	0.063	0.016	0.056	0.056	0.057	0.017	*	32	33
Brooks	0.068	**0.068**	**0.066**	0.069	0.076	**0.083**	**0.075**	**0.072**	0.079	0.083	0.085	*	**4**
Albany Hill	0.070	0.070	0.067	0.070	0.077	0.084	0.076	0.073	0.080	0.084	0.086	0.004	*

Pairwise uncorrected p-values between populations are shown below the diagonal. Numbers in boldface refer to inter-island comparisons. Above diagonal are geographic distances between populations (in km). In this case, values in boldface indicate island to nearest mainland distances.

**Figure 2:**
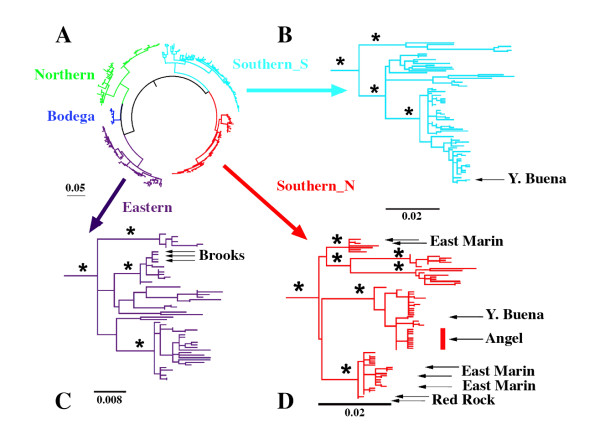
**Phylogenetic relationships among haplotypes found in island populations**. Bayesian phylogram of 282 cytochrome b haplotypes in *B. attenuatus *(A). Colors as in Fig. 1. B, C and D show maximum likelihood phylograms of haplotypes recovered for mtDNA lineages Southern_S, Eastern and Southern_N, respectively. Branch support (Bayesian posterior probabilities >90%) is indicated with asterisks; arrows indicate haplotypes found on island populations.

Phylogenetic analyses performed on datasets composed of all haplotypes for each mtDNA clade plus an outgroup chosen from its sister lineage did not in general resolve well. Some nodes, however, were strongly supported in all analyses (Figure 2). In the Eastern clade, haplotypes found on Brooks Island group with other East Bay haplotypes (Point San Pablo, Pinole Point, Berkeley Hills, Oakland). In the Southern_S clade, haplotypes from Yerba Buena Island cluster with those from the San Francisco Peninsula (Pacifica, San Bruno, San Francisco Watershed, Stanford and Crystal Springs). Moreover, phylogenetic analyses of haplotypes of the Southern_N clade, which is the most widespread and genetically diverse in our dataset, recovered four groups having high support levels. The first of these includes samples from the Point Reyes Peninsula and vicinity. A second includes some haplotypes found in East Marin Island as well as in North Bay localities. A third includes haplotypes from several islands: Yerba Buena, Angel Island, Alcatraz Island, as well as some from the Marin Headlands population in the North Bay and others from the northern end of the San Francisco Peninsula: Presidio and Daly City. Finally, the fourth group includes haplotypes from the North Bay (China Camp, San Rafael, Tiburon, Belvedere, Strawberry Point) as well as haplotypes from the islands of Red Rock and East Marin.

**Figure 3:**
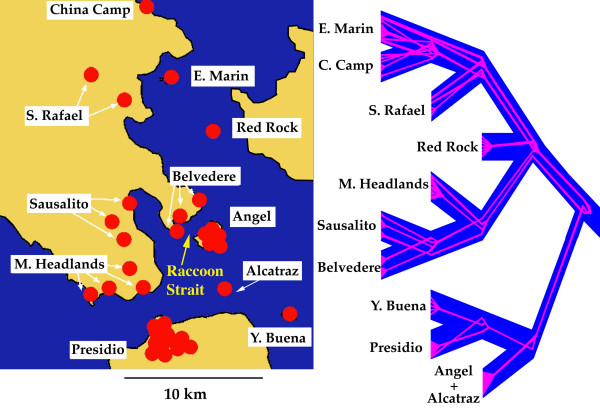
**Population tree and contained gene tree in selected island and mainland populations of *B. attenuatus***. Population tree (blue) depicting relationships between populations in the Southern_N clade (right) and their corresponding locations (left). The underlying gene tree is represented in pink. The yellow arrow indicates the location of Raccoon Strait, separating Angel Island from the mainland. Note that "Belvedere" here includes (from north to south) the localities Bluff Point, Tiburon and Belvedere of Table 1, whereas "Sausalito" includes (from north to south) the populations Strawberry Point, Marin City and Sausalito (grouped on the basis of their proximity for population tree analyses, see text for details).

The program "Mesquite" found 11 population trees that minimized the number of deep coalescences in the best ML gene tree for the pruned Southern_N dataset, including all island as well as reference mainland samples. Figure 3 represents a majority rule consensus of these trees. The following relationships between populations were recovered in all 11 trees: (China Camp+ East Marin), (San Rafael + (China Camp + East Marin)), (Belvedere + Sausalito), (Presidio + Yerba Buena), and (Angel + (Presidio + Yerba Buena). The relationship between Marin Headlands and (Belvedere + Sausalito) was recovered in eight out of 11 trees, whereas the relationship between (Red Rock, Marin Headlands, Belvedere, Sausalito, San Rafael, East Marin and China Camp) was recovered in six out of 11 trees.

Results of BEAST analyses are summarized in Table 3. Median values for estimated time to most recent common ancestors of gene copies (TMRCAs) date from the Pleistocene, with two exceptions: Yerba Buena and East Marin. In the former, the estimated TMRCA of all haplotypes sampled in the island indicates a coalescence time equivalent to that of the common ancestor of all haplotypes in both the Southern_N and Southern_S clades and is therefore, on this measure, much older than the populations of the remaining islands. Indeed, coalescence times for the other islands individually correspond in age to each of these clades separately and are more recent (Pleistocene). The case of East Marin is different, because haplotypes exclusively found on the island are part of two very divergent clades (ISL1 and ISL2, Figure 2 and Table 3), having a common ancestor that may have originated in the Pliocene. Within each of these clades, however, coalescence occurs in the Pleistocene. In other cases, TMRCAs were very similar for islands and their closest mainland populations (Angel *vs *Tiburon and Brooks *vs *Albany Hill, see Table 3). In any case, as indicated above, there are also cases where TMRCAs between island and mainland populations differ substantially (see above and Table 3), making it difficult to extract conclusions about the potential effects of founder events or population sizes in island populations on TMRCA estimates.

**Table 3:** TMRCAs estimated by BEAST for selected groups of samples.

Population	Median (Kya)	95% confidence interval (Kya)
East Marin	1,949	777–3,937
ISL1	68.98	2.05–253
ISL2	364	137–750
China Camp	424	151–786
Red Rock	100	14.85–355
Angel	564	255–1,117
Tiburon	479	156–1,040
Presidio	522	209–1,051
M. Headlands	485	173–1,002
Yerba Buena	6,072	3,137–11,419
Southern_N	95.51	18.2–271
Southern_S	132	33.03–322
Brooks	499	179–1,086
ISL	195	40.28–456
Albany Hill	499	165–1,072

Kya = thousands of years ago. ISL, ISL1, ISL2 refer to groups of haplotypes exclusively found on islands (see text for details). Southern_N and Southern_S refer to haplotypes from these clades found in sympatry on Yerba Buena Island.

### Allozymes

Levels of polymorphism were also high in the allozyme dataset, with values of mean number of alleles per locus ranging from 1.4 in Alcatraz to 2.3 in Presidio and Marin Headlands and percentages of polymorphic loci of 20–45% (Table 4). All loci were in linkage equilibrium and no significant deviations from Hardy-Weinberg equilibrium were found after applying a sequential Bonferroni correction [33] to results of global tests per locus and population, with the exception of locus sMDH (p = 0.0005). This locus was therefore excluded in "Structure" analyses in order to meet assumptions of this program. After removal of this marker, all populations were in Hardy-Weinberg equilibrium.

**Table 4:** Genetic variability in the allozyme dataset.

Population	n	Mean n. alleles	Private alleles	% Pol. loci	H_O_	H_E_
China Camp	70.2	2.2	2	40%	0.184	1.191
East Marin	110.2	1.7	1	40%	0.149	0.145
West Marin	64.9	1.5	-	40%	0.152	0.160
Red Rock	33.0	1.6	-	45%	0.155	0.178
Tiburon	47.8	2.0	3	40%	0.171	0.181
Marin Headlands	88.8	2.3	-	40%	0.166	0.179
Yerba Buena	109.8	1.6	-	40%	0.153	0.165
Alcatraz	103.3	1.4	-	20%	0.071	0.073
Angel	36.6	1.9	3	40%	0.134	0.139
Presidio	134.9	2.3	2	35%	0.139	0.141
Stanford	108.8	1.6	1	25%	0.099	0.100
Crystal Springs	75.7	2.0	5	30%	0.148	0.156
Brooks	55.5	1.5	-	35%	0.073	0.087
Albany Hill	28.8	1.5	-	30%	0.098	0.095

n = mean sample size. Also indicated are mean number of alleles per locus, percentage of polymorphic loci and observed (H_O_) and expected (H_E_) heterozygosities.

**Figure 4:**
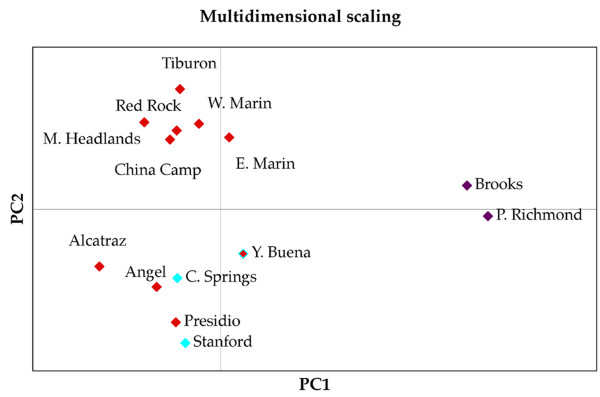
**Cluster analysis of allozyme-based genetic distances between populations studied**. Multidimensional scaling based on Nei's (1972) genetic distances in the allozyme dataset. Populations are colored based on mtDNA haplotypes found in each case. Note that Yerba Buena has mtDNA haplotypes corresponding to two clades.

Figure 4 shows the results of multidimensional scaling based on pairwise Nei's [29] genetic distances. Three clusters are apparent: one composed of the populations of Brooks Island and Albany Hill (East Bay), another includes the mainland populations of the South Bay (Stanford, Crystal Springs, Presidio) plus the islands of Yerba Buena, Alcatraz and Angel; and a third group includes populations in the North Bay (Marin Headlands, Tiburon) as well as the islands of East and West Marin and Red Rock. There is good agreement in pairwise genetic distances between populations based on mtDNA and allozymes (Mantel test, r = 0.711, p = 0.001).

Pairwise F_ST _values between populations are significantly different from zero in all cases (Table 5).

**Table 5:** Genetic differentiation between populations based on allozyme data.

	C. Camp	E Marin	W Marin	R. Rock	Tiburon	M. Headlands	Y. Buena	Alcatraz	Angel	Presidio	Stanford	C. Springs	Brooks	A. Hill
China Camp	*	0.014	0.019	0.021	0.016	0.020	0.044	0.048	0.036	0.056	0.086	0.035	0.109	0.112
E Marin	0.0793	*	**0.011**	**0.024**	0.019	0.029	**0.041**	**0.065**	**0.045**	0.061	0.081	0.039	**0.079**	0.080
W Marin	0.0850	**0.0339**	*	**0.023**	0.014	0.039	**0.065**	**0.066**	**0.058**	0.067	0.080	0.051	**0.101**	0.105
R. Rock	0.0859	**0.0981**	**0.1153**	*	0.018	0.017	**0.050**	**0.076**	**0.053**	0.066	0.074	0.041	**0.112**	0.118
Tiburon	0.0659	0.1070	0.0766	0.0484	*	0.033	0.069	0.084	0.072	0.084	0.099	0.060	0.124	0.122
M. Headlands	0.0977	0.1236	0.1757	0.0777	0.1165	*	0.048	0.072	0.055	0.078	0.100	0.045	0.138	0.142
Y. Buena	0.1593	**0.1359**	**0.1784**	**0.1626**	0.1866	0.2078	*	**0.079**	**0.032**	0.040	0.050	0.013	**0.079**	0.083
Alcatraz	0.1798	**0.2671**	**0.1924**	**0.3500**	0.2690	0.3478	**0.3945**	*	**0.027**	0.048	0.065	0.045	**0.152**	0.180
Angel	0.0990	**0.1507**	**0.1348**	**0.1791**	0.1791	0.2363	**0.1572**	**0.2108**	*	0.011	0.040	0.014	**0.111**	0.126
Presidio	0.2142	0.2254	0.1767	0.2424	0.2451	0.3374	0.2155	0.2930	0.0634	*	0.022	0.014	0.110	0.124
Stanford	0.3414	0.3219	0.2532	0.3077	0.3102	0.4290	0.2789	0.4227	0.2662	0.1597	*	0.021	0.114	0.140
C. Springs	0.0986	0.0845	0.0749	0.0996	0.1137	0.1931	0.0538	0.2717	0.0683	0.0937	0.1489	*	0.099	0.112
Brooks	0.3195	**0.2375**	**0.2805**	**0.3812**	0.3817	0.3969	**0.2487**	**0.5264**	**0.3059**	0.2612	0.3249	0.1919	*	0.005
A. Hill	0.2644	0.1862	0.2409	0.3096	0.3223	0.3358	0.1756	0.5036	0.2256	0.2125	0.3182	0.1374	0.0223	*

Pairwise F_ST _values between populations are shown below the diagonal. All F_ST _values are significantly different from zero. Above diagonal are values of Nei's (1972) genetic distance between pairs of populations. Numbers in boldface refer to inter-island comparisons.

The results of "Structure" analyses suggest that *K *= 3 is the minimum number of clusters that best represents the optimum population structuring in our dataset. The three clusters correspond to three main groups of populations: 1) North Bay populations (populations with mtDNA of the Southern_N clade of Martínez-Solano *et al*., 2007 [4]); 2) East Bay populations (populations with mtDNA of the Eastern clade in Martínez-Solano *et al*., 2007 [4]); and 3) San Francisco Peninsula populations (which include individuals with mtDNA from both the Southern_N and Southern_S clades in Martínez-Solano *et al*. (2007) [4]. Assignment probabilities of each population to the three clusters are shown in Table 6. Cluster 1 includes populations in the North Bay (China Camp, Tiburon and Marin Headlands) plus the East and West Marin islands and Red Rock. The second cluster includes populations bearing mtDNA markers from the Eastern lineage in Martínez-Solano *et al*. (2007) [4]: Brooks Island and Albany Hill. These populations are consistently recovered as distinct clusters in most analyses, with assignment probabilities >85% in all cases. The third cluster includes populations from the San Francisco Peninsula: Presidio, Stanford and Crystal Springs as well as Alcatraz and Angel islands. This group includes populations with mtDNA from the Southern_N and Southern_S clades in Martínez-Solano *et al*. (2007) [4], although, as indicated above, haplotypes from these clades only occur simultaneously in the population of Yerba Buena island. The population from Yerba Buena had assignment probabilities <70% to any of the three clusters, although surprisingly the highest values (62.3%) were associated with cluster 2 (East Bay). Other populations with similar admixture values are East Marin and Crystal Springs (Table 6). In both cases the populations involved are geographically close to areas where major transitions in mtDNA are found.

**Table 6:** Averaged assignment probabilities for individuals in each population to the three inferred clusters in "Structure" analyses.

Population	Cluster 1	Cluster 2	Cluster 3
China Camp (n = 68)	0.750	0.167	0.083
East Marin (n = 32)	0.570	0.327	0.104
West Marin (n = 57)	0.762	0.135	0.102
Red Rock (n = 33)	0.738	0.187	0.074
Tiburon (n = 46)	0.859	0.086	0.055
Marin Headlands (n = 88)	0.846	0.089	0.065
Yerba Buena (n = 104)	0.149	0.623	0.228
Alcatraz (n = 103)	0.027	0.052	0.921
Angel (n = 21)	0.167	0.097	0.736
Presidio (n = 132)	0.178	0.065	0.757
Stanford (n = 108)	0.063	0.063	0.875
Crystal Springs (n = 74)	0.224	0.175	0.601
Brooks (n = 55)	0.024	0.960	0.016
Albany Hill (n = 28)	0.035	0.953	0.012

The three clusters correspond to three main groups of populations: 1) North Bay populations; 2) East Bay populations; and 3) San Francisco Peninsula populations (see text for details). Results are based on the run with the highest likelihood for *K *= 3 clusters. n = sample size.

A feature of the allozyme data that is in contrast to that of the mtDNA findings for the island populations is the infrequent occurrence of unique characters. Whereas each island has one or more unique mtDNA haplotypes (Alcatraz Island excepted), private alleles, that is, alleles that occur on a single island at very low frequency, are less common than in the mainland samples (Table 4). There are three private alleles for the Angel Island population (loci LDH-A, PGM and AK) and one for East Marin Island (GPI). Among the seven mainland localities sampled there are 14 private alleles: five in Crystal Springs (sMDH, LDH-B, PGDH, AAT-1 and SORDH), three in Tiburon (PGM, PGDH and AAT-1), two in China Camp (GPI and PGM) and Presidio (PGDH and GPDH) and one in Stanford (GPI). Conversely, where two or more alleles at a locus occur at moderate to high frequencies, these alleles are typically found throughout the study region in all populations, both island and mainland.

## Discussion

Seawater is generally considered to be an effective barrier retarding dispersal in amphibians. However, recent studies have shown that rafting is the only possible explanation for the presence of certain frog species on oceanic islands far removed from any mainland source [34]. In the case of *Batrachoseps attenuatus*, we have collected them at the high tide mark in both East Marin and Red Rock Islands, indicating some degree of salt tolerance in this species (see [3], for a thorough discussion of this topic). As suggested in that study, the genetic composition of populations established by waifs would differ considerably from that observed in mainland populations that were subsequently isolated by rising sea levels in that genetic resources in the former would represent a highly selected and limited portion of the gene pool of the parental mainland population [3]. Nevertheless, on none of the San Francisco Bay islands is there any evidence from our data for the establishment of *Batrachoseps *populations by means of rafting. Indeed, the high levels of genetic variability occurring on most of the islands tend to argue against this hypothesis. However, in the unlikely event (because of the observed numbers of exclusive haplotypes found on islands) that rafting was a common occurrence, distinguishing between the two hypotheses could become problematic because the impact of founder effects would be diminished.

The patterns of genetic variation observed in slender salamander populations on the San Francisco Bay islands, using both mitochondrial and nuclear markers, indicate an early presence of the salamanders in the area rather than recent colonizations. Levels of genetic diversity are high, and in most islands exclusive mtDNA haplotypes were found. Where island and mainland localities share haplotypes the mainland populations are usually those that are geographically closest to the island (Table 2). Estimated TMRCAs for island haplotypes clearly predate the timescales associated with the formation of the Bay, 9,000 years ago. However, these estimates reflect gene divergence and thus necessarily predate the actual time of the start of population divergence, although it seems safe to hypothesize that populations were already established on the hilltops that later became islands, probably before the last interglacial period. Further, there was probably a significant pattern of genetic structuring prior to the formation of the Bay as we know it today.

**Figure 5:**
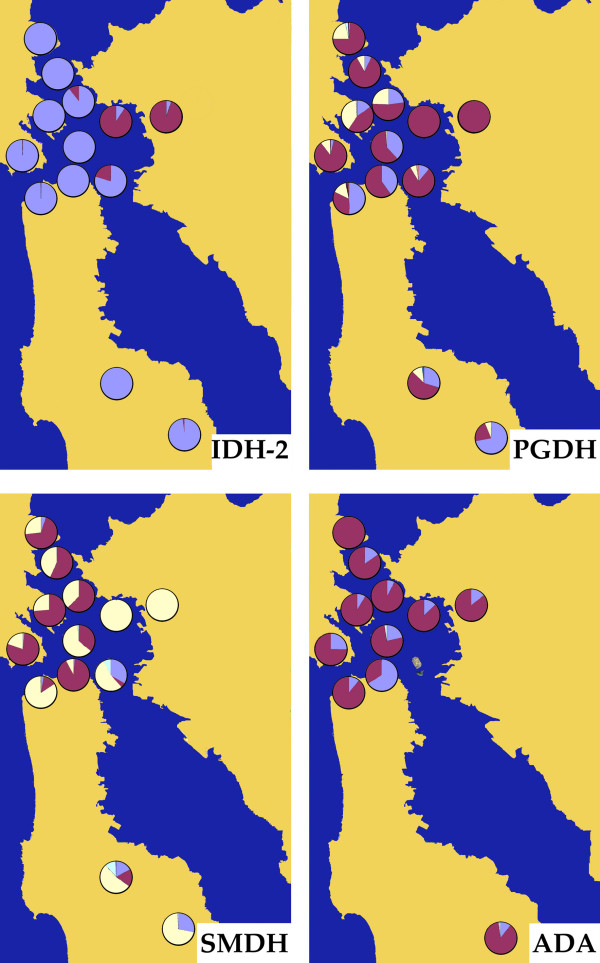
**Some representative patterns of allozyme variation in the study area**. Allelic frequencies scored in each population for loci IDH-2, PGDH, SMDH and ADA. Note that no data were available for ADA in Yerba Buena Island and Crystal Springs. Population locations as in Figure 1.

There are exceptions to this pattern of early establishment of differentiated populations: in the case of Alcatraz Island, we found only a single mtDNA haplotype occurring in the 30 individuals sampled, with this haplotype also occurring on Angel Island. In addition, levels of genetic diversity in the allozyme data for Alcatraz Island are also strikingly the lowest among the Bay islands (1.4 alleles per locus, 20% polymorphic loci, observed heterozygosity = 0.071, Table 4), and genetic distances are lowest with respect to Angel Island (D_Nei _= 0.027). These findings are consistent with a recent introduction involving individuals from Angel Island, in keeping with the proposal of previous studies [1]. This introduction event could have taken place when land was taken from Angel Island to level Alcatraz prior to construction of the prison [1]. In the allozyme data, the fact that the same alleles are observed in both populations, but sometimes in very different frequencies suggests the existence of a founder effect in the Alcatraz population. This pattern is evident, for instance, in the sMDH and ADA loci (Figure 5). Genetic signals of a founder effect would be expected if the hypothesis of a recent, anthropogenic introduction is correct, and they might also explain why the Alcatraz population stands as genetically distinct in some analyses, as reflected in high F_ST _values (Table 5).

With respect to the evolutionary history of salamander populations in the study region, data derived from both mitochondrial and nuclear genes support the existence of three distinct evolutionary units, although there are discordances with respect to their individual limits. The two major barriers identified by both mtDNA and allozymes seem to be associated with the Colma and Raccoon Straits (Figs. 1 and 3, see below). According to mtDNA data, three mtDNA clades coexist in close proximity in the region, with the Southern_N clade being the most widespread and present in most of the island populations. The deepest split between mtDNA haplotypes is that between the Eastern and the Southern (including both the Southern_S and Southern_N) clades, which might date as far back as the Miocene [4]. This pronounced break is also apparent in the allozyme data and is apparently unrelated to any present geographic feature. Allelic frequencies at some loci are markedly different in the Eastern populations of Brooks Island and Albany Hill with respect to other populations (for example, in loci IDH-2 and PGDH, Figure 5). Higher genetic distances are observed in comparisons between the two Eastern populations and the remaining populations than in any other comparison (Table 5). Results from "Structure" analyses recover the two Eastern populations as a distinct cluster for most values of *K *(all analyses with *K *= 3 to *K *= 8), with high assignment probabilities in all cases, also highlighting the genetic distinctiveness of these populations among the total. This is in agreement with Anderson's [3] conclusions regarding morphological differentiation of island populations with respect to the mainland.

Morphometric variables studied by Anderson (1960) [3] included: snout-vent length, tail length, head width, limb length, number of vomerine teeth and number of costal grooves. His results indicated mainland populations grouped into two main types: a general "mainland" type exemplified by populations in Marin Peninsula, Sonoma and San Mateo counties, and a second group including the populations of Berkeley and Point Richmond, in the East Bay. He found no evidence for an "island type". Instead, he found the populations of Red Rock and Brooks Island to be most differentiated morphologically from mainland populations and from each other. But the Brooks Island population was more similar to the Point Richmond and Berkeley populations in his sample, whereas that on Red Rock showed affinities to Angel Island and East Marin in body proportions. This pattern of morphological variation is totally concordant with genetic data and thus the observed similarities between populations probably reflect a shared evolutionary history rather than the effect of similar adaptive factors associated with evolution on the highly simplified island environments [3].

The observed high genetic distances between the five mtDNA clades identified in a previous study [4] raised the possibility that *B. attenuatus *is in fact a complex of cryptic species. However, values of D_Nei _between the Eastern populations from Brooks Island and Albany Hill and the remaining populations range from 0.079–0.142 (the comparisons involving Alcatraz are outlier values of D_Nei _= 0.152–0.180, perhaps related to the existence of a founder effect in this population, see above and Table 5). These values are within the range observed in intraspecific studies [35], suggesting that gene flow and balancing selection for different alleles are major forces maintaining genetic cohesion in *B. attenuatus *across very divergent mtDNA boundaries.

Both the Eastern and the Southern_N clades show much higher values of genetic diversity in populations north of the San Francisco Bay, suggesting colonization of today's islands as well as the San Francisco Peninsula was a comparatively recent event. This is evident in the relatively low levels of haplotypic variation observed in the Presidio population with respect to Marin County populations (Table 1). On the contrary, the Southern_S clade shows much higher levels of genetic diversity at the southern end of its range, near the Santa Cruz Mountains, and it seems likely that their establishment at the southern end of the San Francisco Peninsula is also a recent event. The sharp mtDNA break between the Southern_N and Southern_S clades is correlated with the geological feature known as the Colma Strait, a sea pass which was the main drainage of the Bay before the opening of the Golden Gate. The Colma Strait was open at a minimum between 570,000 and 125,000 years ago [36], and probably had a major effect as a barrier to dispersal in slender salamanders. Some authors have suggested that during the last interglacial, 125,000 years ago, Marin County and the San Francisco Peninsula north of the Colma Strait were connected [36], which would have allowed colonization of this area by the Southern_N clade at this time while preventing gene flow between mtDNA clades Southern_S and Southern_N. However, this sharp break in mtDNA haplotype distribution between the Southern_N and Southern_S clades is not evident in the allozyme dataset, where the two groups that are consistently recovered in different analyses correspond to populations north of the Bay (plus the Marin Islands and Red Rock) on the one hand and populations in the San Francisco Peninsula (plus Angel Island, Alcatraz and the admixed Yerba Buena population) on the other (Figures 3 and 4). This suggests extensive gene flow across mtDNA boundaries following secondary contact after the Colma barrier ceased to exist. In contrast, this allozyme split seems to be related to the present configuration of the drainage of the Sacramento-San Joaquin rivers, with the two deepest channels in the Bay being Raccoon Strait (separating Angel Island from the formerly connected Tiburon Peninsula) and the Golden Gate (separating Marin County from the San Francisco Peninsula) (Fig. 3) [37].

An interesting pattern that demands further explanation is that observed on Yerba Buena Island. On the one hand, notwithstanding our extensive sampling, it remains the only instance from across the species range where we found coexistence of salamanders of two different mtDNA lineages. However, in addition to the haplotypes from both clades occurring on the island there are as well mainland haplotypes that are also found in the geographically closest mainland populations (Presidio and Pacifica), suggesting that this contact zone was established naturally rather than being related to anthropogenic introductions. However, the allozyme data for the Yerba Buena population suggest a complex history with discordant pictures derived from the mitochondrial and nuclear data. This population stands out from others as genetically distinct, and although "Structure" analyses suggest that this is clearly an admixed population with assignment probabilities to inferred clusters always <70%, some analyses, including the preferred structuring into three clusters, indicate a significant genetic input from populations in the Eastern clade (Table 6). This is surprising because not a single mtDNA haplotype of the Eastern clade was observed in our sample. However, because we did not use the same samples for mtDNA and allozyme analyses, it remains to be tested whether this is an artifact of the sampling or whether the contact zone actually involves the meeting of three rather than two mtDNA lineages, with one of them having been extirpated from the contact zone. The fact that salamanders of the two haplotype lineages occur in microsympatry and that the allozyme data show a close correspondence between observed and expected heterozygote classes and overall heterozygosities (see Table 4) suggests that currently the Yerba Buena *Batrachoseps *population is panmictic. Another, perhaps more plausible explanation for the presence of Eastern clade allozyme characters on Yerba Buena Island is recent anthropogenic introduction. The totally man-made artificial island, known as Treasure Island, which is narrowly connected to Yerba Buena Island was constructed in part of rock quarried on Brooks Island. Anderson [3] considered Yerba Buena Island to be the most likely of those he studied to have had its salamander population influenced by introductions from the outside and cited the decades of house building and extensive landscaping as producing the conditions for the introduction of *Batrachoseps*, possibly from multiple sources. None-the-less, if this explanation is accepted, the absence of an mtDNA Eastern haplotype is puzzling.

Because of their small size, West Marin and Red Rock Islands are the only two among the Bay islands on which initial *Batrachoseps *populations may have been small enough to have had their gene pools affected by detectable genetic drift. East Marin and West Marin Islands are in close proximity, the channel separating them being only 170 m. wide and of shallow depth. It seems probable that prior to their isolation from each other, and notwithstanding the low vagility of *Batrachoseps*, the two populations would have constituted a single deme. This conjecture is bolstered by analyses of the allozyme data, where values for pairwise Nei's genetic distances and F_ST _are, excepting those separating Brooks Island and Albany Hill, the lowest in the dataset (Table 5) and allele frequencies at single loci are similar for the two islands (data not shown).

The Red Rock Island *Batrachoseps *population presents a different genetic picture. The closest mainland is to the east, where genetic characteristics correspond to the East Bay clade, typified by Brooks Island, Point Richmond, Albany Hill, Oakland and Berkeley Hills populations. Anderson [3], in his study of the Red Rock population using morphometric and meristic characters found that the means for each of these characters were most similar to those of the west side of the Bay, typified by Marin County and the San Francisco Peninsula, rather than those of the East Bay. Moreover, means for these character states were the most diverged of all populations and had deviations from the mean strikingly lower than all other populations. These findings were interpreted to indicate that the Red Rock population was founded by waif dispersal, thereby resulting in a much-reduced gene pool for this founder population [3]. Strong freshwater currents from the San Joaquin – Sacramento Delta to the north and tidal currents from the South Bay along with experimental evidence showing that gravid female *Batrachoseps *are capable of surviving a period of rafting are cited as additional evidence in support of this hypothesis [3]. Further, the high incidence of tail autotomy in the Red Rock *Batrachoseps *population was conjectured to be the result of heavy predation by the salamander *Aneides lugubris*, another factor which, together with competition for spatial resources with the same species, have been suggested to have influenced the fast evolution of morphological differences by keeping the population small enough for genetic drift to occur [3,38].

In contrast, if waif dispersal is the source of the Red Rock *Batrachoseps *population, it seems more likely that the strong tidal current through the Golden Gate sweeping the shores of the Marin Headlands and the northern end of the San Francisco Peninsula would be the mediator and would account for the similarity between these populations and that of Red Rock Island. However, regardless of tides and currents, our data suggest a different scenario for the origin of the Red Rock Island *Batrachoseps *population. In a sampling of only seven animals, two unique mtDNA haplotypes were found. This is comparable with all other island populations save that of Alcatraz (Table 1). In addition, allelic diversity found in our allozyme data (Table 4) is not reduced relative to other populations, again excepting Alcatraz. This argues against the founding of the Red Rock population by one or a few waifs. Rather, the genetic marker characteristics of this population suggest the isolation of a pre-existing population typical of the Southern_N clade [4] as the more plausible hypothesis.

## Conclusion

In summary, our study provides a general picture of the results of 9,000 years of independent evolution of salamander populations on at least four of the islands in San Francisco Bay: East Marin, Red Rock, Angel and Brooks Island. Additionally, signal of older historical events that predate island formation is also prominent in our dataset; genetic and morphometric data delineate well-differentiated lineages that diverged from a common ancestor in the Miocene (Southern_N *vs*. Eastern, see [4]). These lineages meet in the proximity of San Francisco Bay and are present on different islands (East Marin, Red Rock and Angel: Southern_N; Brooks: Eastern). This indicates that, contrary to findings in other taxa from areas that were heavily affected by Quaternary glaciations, salamander populations were genetically structured well before the Bay islands were formed during the Holocene, and therefore the observed phylogeographic structure is old, rather than a shallow, transient signal produced by recent stochastic factors [39]. In addition, the origin of haplotypes that are exclusively found on certain islands exceeds considerably the age of those islands and therefore those haplotypes probably evolved *in situ *wellbefore sea levels rose. Thus, from a genetic standpoint it can be said that the last 9,000 years of evolution on these islands have been characterized by relative stability, with the occasional extinction of some haplotypes or alleles that were formerly shared between island and mainland populations but overall maintaining high levels of variation (with the noted exception of Alcatraz). In contrast, there is some evidence for rapid morphological changes between populations in some islands and their closest mainland counterparts (Red Rock *vs*. Marin County populations, Brooks Island *vs*. Point Richmond) [3]. This pattern of rapid morphological divergence (e. g., resulting from founder effects) is similar to that observed in other studies of recent colonization of island habitats [40-43].

## Authors' contributions

IMS collected samples, compiled the mtDNA dataset and analyzed both datasets. RL collected samples and compiled the allozyme dataset. Both authors wrote the manuscript, read and approved the final version of the manuscript.

## Supplementary Material

Additional file 1 **Appendix 1.** Sampling localities, including mtDNA clade, latitude and longitude coordinates, sample sizes and voucher numbers. Click here for file

Additional file 2 **Appendix 2.** List of enzymes surveyed. EC = Enzyme Commission. Click here for file
